# MINDPRES: A Hybrid Prototype System for Comprehensive Data Protection in the User Layer of the Mobile Cloud

**DOI:** 10.3390/s25030670

**Published:** 2025-01-23

**Authors:** Noah Oghenefego Ogwara, Krassie Petrova, Mee Loong (Bobby) Yang, Stephen G. MacDonell

**Affiliations:** 1 Department of Computer and Information Sciences, School of Engineering, Computer and Mathematical Sciences, Auckland University of Technology, City Campus, Auckland 1010, New Zealand; fego.ogwara@macvad.com (N.O.O.);; 2 Centre for Data Science and AI, School of Engineering and Computer Science, Faculty of Science and Engineering, Victoria University of Technology, Kelburn Campus, Wellington 6012, New Zealand; stephen.macdonell@vuw.ac.nz

**Keywords:** mobile device security, android OS, malicious app, app behavior, machine learning model, permissions, intents, risk assessment, intrusion detection, static analysis, hybrid analysis

## Abstract

Mobile cloud computing (MCC) is a technological paradigm for providing services to mobile device (MD) users. A compromised MD may cause harm to both its user and to other MCC customers. This study explores the use of machine learning (ML) models and stochastic methods for the protection of Android MDs connected to the mobile cloud. To test the validity and feasibility of the proposed models and methods, the study adopted a proof-of-concept approach and developed a prototype system named MINDPRESS. The static component of MINDPRES assesses the risk of the apps installed on the MD. It uses a device-based ML model for static feature analysis and a cloud-based stochastic risk evaluator. The device-based hybrid component of MINDPRES monitors app behavior in real time. It deploys two ML models and functions as an intrusion detection and prevention system (IDPS). The performance evaluation results of the prototype showed that the accuracy achieved by the methods for static and hybrid risk evaluation compared well with results reported in recent work. Power consumption data indicated that MINDPRES did not create an overload. This study contributes a feasible and scalable framework for building distributed systems for the protection of the data and devices of MCC customers.

## 1. Introduction

The significant amount of data transferred through the mobile Internet has attracted significant attacker attention. Increased user concerns about the security of their data have the potential to slow down the adoption of the services offered by cloud computing (CC) and mobile cloud computing (MCC) service providers [1,2,3]. Intrusion detection and prevention play a critical role in protecting user data from malware.

The three main layers of the MCC architecture are the user layer (UL), the communication layer (CL), and the cloud service provider layer (CSPL). All three layers are vulnerable to attacks that can lead to data privacy breaches, data loss, or malicious data replication [4,5,6]. However, the effective protection of the MD and MD user data is of particular importance [7,8]. First, the CC data security issues related to multi-tenancy and virtualization affect the information stored in the MCC infrastructure [9,10]. Second, when using cloud-based computation resources for executing resource-demanding applications (apps), MD users connect to a cloud or an edge server and may upload data for further processing [11]. A malicious app active on a user’s MDs may breach the security of the data stored in the mobile cloud [12,13] and disrupt the services of mobile cloud apps in critical areas such as education, banking, and healthcare [14,15,16].

Malicious apps on MDs are a significant data security and privacy threat [17,18,19,20]. Therefore, it is necessary to develop effective approaches for malicious app detection to mitigate the risks to the reliability of the MCC environment [5]. However, traditional detection methods have difficulty keeping up with new threats and attacks, such as repackaging a popular app with a malicious payload [21,22,23,24,25,26]. New approaches such as machine learning (ML) models can be more effective in detecting threats posed by malware that is evolving and becoming much more sophisticated.

The development and implementation of ML-based detection techniques is challenging due to the computational and energy constraints of the MDs, and in some cases, the need to modify the kernel of the device operating system (OS). If some of the defensive mechanisms can be moved to a cloud or an edge server, this would increase the feasibility of implementing a comprehensive defense system for MD protection [27,28,29,30].

### 1.1. Related Work

Researchers in the area have used both static and dynamic app analysis as a means of malware detection. Earlier work predominantly deployed a static approach, commonly using app permissions extracted from the app’s Android package kit (APK) file as classification features [31]. With cybercriminal attacks becoming more sophisticated, the focus has shifted toward the dynamic analysis of features representing app behavior. For example, the system proposed by Ribeiro et al. [18] monitors MD behavioral characteristics such as network traffic and CPU, battery, and memory use to detect unusual patterns. Zhou et al. [19] proposed a dynamic ML model for malicious attack detection; the model used a large set of 166 dynamically extracted features. The detection engine acquires run-time system calls from unknown Android apps to create an input feature vector table. The ML model classifies an app as benign or malicious based on the table. Bhat et al. [32] and Roy et al. [33] also explored dynamic features. Both studies tested extensively several ML models by applying them to different datasets; system calls, binder calls, and network traffic data were used as dynamic input features.

Work on improving the performance of static ML detection models also continued. For example, Jannath et al. [17] proposed an ontology-based intelligent model using an ML classifier trained on app permissions. Alazab et al. [34] developed a static malware detection model using app permissions and application programming interface (API) calls; they sought to identify a set of best-performing strategies for selecting relevant API calls. Kirubavathi et al. [35] applied a static approach to build a model specifically for ransomware detection. The model used a relatively small set of 22 static features (permissions). Aljarrah et al. [36] also used static analysis, adding a set of contextual use features (services, receivers, activities, and providers) to the set of 50 other static features (including permissions and API calls). Mahindru et al. [37] applied a static approach that used permissions and API calls as features, and app ratings and the number of app downloads as additional features.

Hybrid approaches that combined static and dynamic analysis methods also gained attention, especially in recent years. For example, Maryam et al. [38] proposed using app permissions and intents as static features and information leakage, cryptography’s exploitation, and network manipulations as dynamic features. Their experimental results indicated that the best classification results were achieved when the static and dynamic features were analyzed simultaneously. Shatnawi et al. [39] also applied a hybrid approach. Permissions extracted from the APK files and dynamic features extracted from running the apps in a controlled environment were used as static and dynamic features, respectively. Their model focused on action repetitions (the number of activities representing normal or suspicious data access activities). Aldhaferi et al. [40] trained a support vector regression (SVR) classifier for hybrid analysis on a set of 327 features. The static features were extracted from the APK files and included permissions and other metadata; the dynamic features (system calls, API calls, network traffic) were obtained by executing the APK files in a controlled environment.

Miltenberger et al. [41] developed AppRunner, a flexible and extensible architecture for hybrid app analysis. AppRunner facilitates hybrid app analysis in a test environment. It implements app instrumentation (i.e., adding code to the app software) to obtain behavioral data while the app is running. Furthermore, Surendran et al. [42] assumed the existence of interdependencies between static and dynamic features and proposed a two-stage probabilistic ML model. At the first stage, three linear regression (LR) classifiers produce classification outputs based on static and dynamic features (permissions, API calls, system calls). At the second stage, a tree-augmented Naïve Bayes (NB) model interprets the conditional dependences between the outputs as a tree structure and provides a final classification.

Several studies have developed deep learning (DL) models for app malware detection. For example, Obidiagha et al. [43] proposed the hybrid DL model DeepImageDroid that combined a convolutional neural network (CNN) and a visual transformer. The model converts the binary feature values to a grayscale image; the image is fed to an ensemble DL. Once trained on static features, the models can be deployed within a real-time monitoring system. Asmitha et al. [44] created a multilayer architecture comprising both ML and DL algorithms. Similarly to other studies, the authors used static features (permissions, activities, services, receivers) extracted from the APK files. The dynamic features (system calls) were extracted by running the APK files in an emulator. Sonya et al. [45] proposed a hybrid system for ransomware detection in which the static features (e.g., permissions) were extracted from the APK files. The dynamic features (e.g., API calls) were extracted by running the apps in a sandbox or an emulator.

In another study with a specific focus [46], the authors deployed a range of DL classifiers to investigate their effectiveness in detecting crypto-mining malware. The hybrid system used opcodes and system calls as static and dynamic features, respectively. The dynamic features were extracted in an emulated environment. In contrast, Alzaylaee et al. [47] tested their hybrid DL model on apps installed on MDs. The MDs were connected to a dynamic feature extraction system. Permissions were used as static features (extracted before running the app). According to the authors, using an actual MD rather than an emulator creates a more realistic controlled testbed.

Most ML/DL malware detection modes are trained on well-known datasets. This makes it easier to compare results and identify the best working approaches. However, as malicious actors develop new techniques for evading detection, these datasets ‘age’ quickly [48]. This increases the probability of misclassifying apps whose static and dynamic features have been purposefully designed to obfuscate malicious behavior. As a means of mitigating the risk, several recent studies have explored a graph representation learning (GRL) approach. In GRL, the features representing app behavioral activities (e.g., network communications) are extracted at run time and used to construct a directed graph where the app activities are the nodes. The resulting graph structure is converted to a low-dimensional vector that serves as input to an ML/DL classifier [49].

GRL can be applied statically by creating a function call graph that is based on the app source code. For example, the HGDetector proposed by Feng et al. [50] constructs a node interaction graph that captures dynamic network traffic and a network behavior function call graph that captures static function call features. The two graphs are fused to represent app behavior. The authors experimented with feeding the output of the fusion to several ML classifiers, with the best results obtained by the support vector machine (SVM) model.

Graph neural networks (GNNs) can process graph input by node embedding [49]. For example, Wu et al. [51] proposed the DL model DeepCatra which comprised a bidirectional long short-term memory (LSTM) network and a GNN. The LSTM network captures app behavior features (opcodes) based on potentially dangerous API calls while the GNN constructs an abstract flow graph based on these API calls and on inter-component interactions. The two outputs are merged in a hidden layer which estimates the probability of the app being malicious. Furthermore, the DL model GSE Droid proposed by Gu et al. [52] integrates the two GNN technologies GraphSage and SAGPooling. The model aims to improve the precision of the API call opcode representation, for the fast malicious behavior patterns identification in the API call graph.

Most publicly available training datasets contain a relatively small number of malicious samples compared to the number of benign samples; this reduces the accuracy of the classification. To address the issue, Li et al. [53] proposed a model that deployed an unbalanced heterogeneous graph embedding to uncover hidden relationships amongst app features. The accuracy of detecting malicious app design was improved by using both static app features and the semantic relationships between them. In a related work, Li et al. [54] explored the use of heterogeneous graphs for similarity search and quick discovery of previously unknown malicious apps.

### 1.2. Research Problem and Study Contributions

Most models proposed in past research have been tested in laboratory conditions, using datasets available from various repositories. While dynamic and hybrid models have achieved good classification accuracy and other performance metrics, the extraction of features for dynamic and hybrid analysis involved executing the apps in a controlled environment. This is a particular drawback affecting the implementation of ML model for real-time protection against malicious intrusions. Only a few studies, e.g., refs. [44,54], have considered the feasibility of developing software systems that can be deployed on MDs. A parallel (cloud-host) architecture for malware detection was proposed in [26]; however, it worked with static features only, extracted from the APK files.

Yunmar et al. [48] identify the lack of research in developing usable MD protection systems, and in implementing ML/DL solutions on real MDs as major challenges. Sharma et al. [55] similarly point out that while most approaches proposed in the literature imply testing apps in a specialized setup, MD users urgently need ‘on-device’ models that can generate malware detection reports in real time. The heavy computational load of dynamic and hybrid detection models and the lack of fully automated systems for behavioral data gathering and processing present an additional barrier [10].

This study addresses the problems identified above. It proposes MINDPRES, a scalable hybrid system for the comprehensive protection of an MD connected to the mobile cloud and develops a prototype that demonstrates the feasibility of the methods used. The main contributions of this study are:

- A data-driven, cloud-based method for the static risk assessment of mobile apps resident on an MD.
- An effective, innovative filter-based feature selection (FS) technique for static app classification.
- A proof-of-concept prototype of an innovative distributed hybrid intrusion detection and prevention system (IDPS).
- A scalable framework for building distributed systems for the protection of MCC user data and devices.

The remainder of the paper is organized as follows. Section 2 provides an overview of the functionalities of the prototype system and describes the methods used. The design of MINDPRES and the performance evaluation results are presented in Section 3. In Section 4, we compare our work to relevant results reported in the extant literature, discuss the contributions and limitations of the study, and suggest directions for further research.

## 2. Materials and Methods

**Figure 1:**
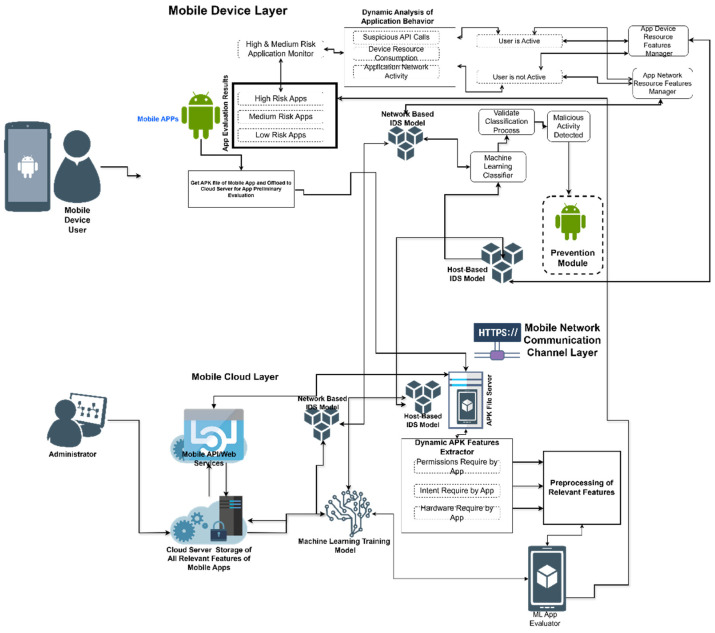
MINDPRES functionalities.

To test the validity and feasibility of the proposed models and methods, this study adopted a proof-of-concept approach and developed a prototype MINDPRES system. The functional overview of the MINDPRES prototype is shown in Figure 1. MINDPRES is a distributed system that has two major building blocks: a cloud-based app evaluator for static analysis and an MD-based IDPS for hybrid analysis.

The app evaluator works with a cloud-based ML model for app classification. It assigns a risk category to each evaluated app based on the features and intents used by the app. The IDPS comprises a host intrusion detection system (IDS), a network IDS, and an intrusion prevention system (IPS). The host IDS uses an ML model for the analysis of static app features (permissions and intents) granted at run time, while the network IDS uses an ML model for the analysis of dynamic app features (run-time network traffic data). The IPS blocks malicious activities detected by the IDPS.

The models used in MINDPRES are trained on a cloud-based server. Once deployed, the system works as follows:

1. At installation time, MINDPRES analyzes each app on the MD and determines its risk category (high, medium, or low). High-risk and medium-risk apps are placed on a watchlist.
2. When a new app is installed, the host IDS is automatically invoked to perform a static analysis and assign the app a risk category.
3. The network IDS and the host IDS monitor app API calls and network activities, including when the MD is idle. The system prioritizes monitoring apps that are already on the watchlist.
4. The IPS automatically blocks apps when suspicious activities or malicious network traffic are detected. MINDPRES deals with possible false alarms by giving the user the option to override the block and execute the app.

To develop the functional capabilities of MIDNPRES we drew on the approaches and experimental results reported in our previous work [18,19,56]. The methods used in this study are described below.

### 2.1. Feature Selection Method

To select the features for static ML analysis, this study uses intrinsic feature dispersion (IFD) as the FS method. IFD is a filter-based feature selection method that was proposed and evaluated in [56]. It takes into consideration the outcomes of the statistical analysis of the use of permissions and intents by the apps represented in the model’s training dataset. The method was developed and tested using a training dataset of 28,306 APK samples (9879 benign and 18,427 malicious) collected from the AndroZoo [57] and RmvDroid [58] repositories. Collectively, the apps in the training dataset had 263 unique features (132 permissions and 131 intents).

The set of potential features was reduced first by excluding features that were likely to be found in the APK files of both benign and malicious apps, such as the permission to use the Internet. Permissions and intents that were rarely used by apps of any type were also excluded. The exclusion/inclusion thresholds were set to 3% and 90%, respectively (i.e., permission or an intent was excluded if less than 3% or more than 90% of the apps in the training dataset used it). The outcome was a permission set M and an intent set N of potential classification features. Next, each permission *M_i_*, *i =* 1, 2 … *m* from the set *M* and each intent *N_j_*, *j =* 1, 2 … *n* from the set N was selected or rejected as an input feature, as described below.

For each permission M*_i_*, we define the measures to represent the relative use of this permission by all benign and all malicious apps in the training dataset as

(1) $$\xi_{i}=\frac{B\left(M_{i}\right)}{G\;}\times 100\%$$

And

(2) $$\eta_{i}=\frac{L\left(M_{i}\right)}{G\;}\times 100\%$$

where G is the number of benign apps in the training dataset, *B*(*M_i_*), *i =* 1, 2 … *m* is the number of benign apps in the training dataset using permission *Mi*, *U* is the number of malicious apps in the training dataset, and *L*(*M_i_*), i = 1, 2 … *m* is the number of malicious apps in the training dataset using permission M*i*.

The permission selection function *E*(*M_i_*) is defined as

(3) $$E\left(M_{i}\right)=\left\{\begin{array}{c} 1,\;\;if\frac{\delta_{i}}{min\left(\xi_{i},\eta_{i}\right)}>\frac{\varepsilon_{i}}{2}\\ 0,\;\;if\frac{\delta_{i}}{min\left(\xi_{i},\eta_{i}\right)}\leq \frac{\varepsilon_{i}}{2} \end{array}\right.$$

where

(4) $$\varepsilon_{i}=min\frac{\left(\xi_{i},\eta_{i}\right)}{max\left(\xi_{i},\eta_{i}\right)}$$

and

(5) $$\delta_{i}\left(\xi_{i},\eta_{i}\right)=\left|\xi_{i}-\eta_{i}\right|=\left\{\begin{array}{c} \xi_{i}-\eta_{i},\xi_{i}\geq \eta_{i}\\ \eta_{i}-\xi_{i},\wedge \xi_{i}<\eta_{i} \end{array}\right.$$

A permission *M*_i_ was selected as a feature if E(*M*_i_) returned a value of 1. Intents were selected similarly. Table 1 shows the 39 unique features selected: 30 permissions (features *S_1_–S_39_*) and nine intents (features *S_31_–S_39_*).

### 2.2. Ensemble ML Model for Static Analysis

**Figure 2:**
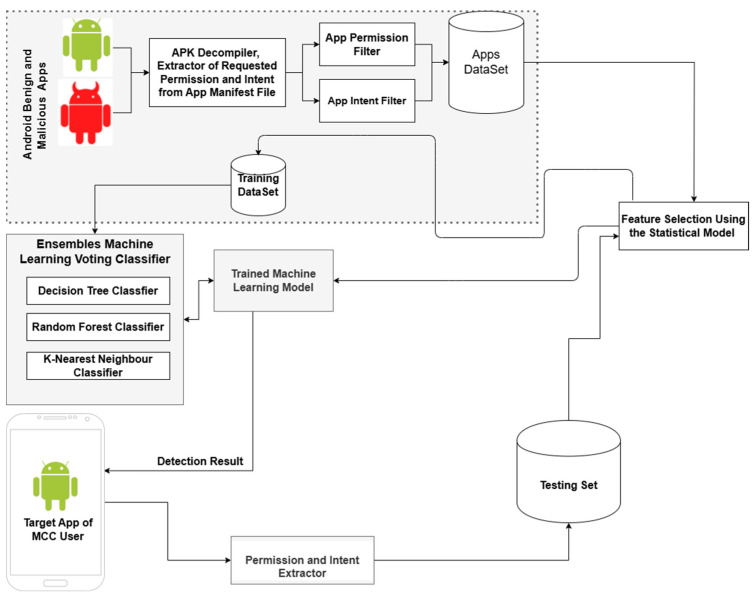
Ensemble ML model for static analysis.

The 39 features selected by applying the IDF method (Section 2.1) were used to train an ensemble ML model for static app classification (Figure 2).

Ten ML classification algorithms were considered for the ensemble: Decision Tree (DT), Random Forest (RF), AdaBoost, NB, Stochastic Dual Coordinate Ascent, Multi-layer Perceptron, K-nearest Neighbors (KNN), Linear Discriminant Analysis, LR, and SVM. These algorithms are well suited for a classification problem such as predicting an app as malicious or benign and have been widely used in related empirical work [31,59,60]. We experimented to compare the performance of the ML algorithms and build an effective ensemble ML classifier. We used the ML Scikit library implemented using the Python programming language running on a remote virtual machine with the following hardware configuration: AMD 32-Core CPU @ 2.20 GHz (2 processors) (Advanced Micro Devices, Inc. (AMD) Sunnyvale, CA, USA), 64 GB RAM, and a 500 GB hard disk drive. The experimental models worked with the features in Table 1 and were trained using the training dataset that was used to identify these features. We tested the models with a test dataset that contained 2001 benign samples and 3661 malicious samples.

When comparing classification accuracy (CA), precision rate (PR), error rate (ER), and false alarm rate (FAR), the three best-performing classifiers were DT, RF, and KNN. These classifiers were selected for the majority-voting ensemble ML. A comparison of the performance of the models is presented in Table 2. It shows that the ensemble model performed better than the other models in several important metrics. For example, it achieved the highest CA of 98.13%, the highest F1-score measure (FM) of 98.55%, the highest PR of 98.82%, the lowest ER of 1.87%, and the lowest false positive rate (FPR) of 2.15%. The FAR of 1.93% achieved by the ensemble model was also the lowest and was significantly lower than the FAR of the other three models.

### 2.3. Static App Risk Evaluation Method

The static method for assigning a risk category to an app was proposed and tested on a small sample of apps in [19]. The app risk category (low risk, medium risk, high risk) is determined based on two inputs: (i) the app’s classification as benign or malicious by the ensemble ML model for static app classification described in Section 2.2, and (ii) the calculated value of the app’s risk score. The risk score reflects the app’s use of a specific reference set of dangerous permissions.

#### 2.3.1. Risk Scores of the Dangerous Permissions in the Reference Set

The dangerous permissions in the reference set represent the ‘topmost dangerous permissions’ that are commonly used by apps likely to be found on user devices. Similarly to [61], we assume that an app that uses a higher number of topmost dangerous permissions is more likely to be malicious compared to an app that uses a smaller number of them. This study uses the 15 topmost dangerous permissions identified in [19] as a reference set of dangerous permissions (Table 3).

For each dangerous permission *P_i_*, *i* = 1, 2, … 15, its risk score r(*i*) is calculated as

(6) $$r(i)=\sqrt{\alpha_{i}-\alpha_{i}\beta_{i}}$$

where

(7) $$\alpha_{i}=\frac{L\left(Pi\right)}{U}$$

and

(8) $$\beta_{i}=\frac{B\left(Pi\right)}{G}$$

Here, *B*(*P_i_*), *i =* 1, 2, … 15 is the number of benign apps in the training dataset using dangerous permission *Pi*, and *L*(*Pi*), *i* = 1, 2, … 15 is the number of malicious apps in the training dataset using dangerous permission *Pi.*

#### 2.3.2. App Risk Score

An app *a* that is resident on an MD may be using one or more of the dangerous permission P*_i_*, *i* = 1, 2, … 15. Let $\lambda \left(a,i\right),\;i=1,\;2,\ldots 15$ equal 1 if the app uses dangerous permission *P_i_* and 0 otherwise. The app’s risk score *R*(*a*) is a value in the interval [0, 1] which is calculated as

(9) $$R\left(a\right)=\frac{1}{k}\overset{n}{\underset{i=1}{\sum }}\lambda \left(a,i\right)r\left(i\right)$$

where

(10) $$k\left(a\right)=\overset{n}{\underset{i=1}{\sum }}\lambda \left(a,i\right)$$

#### 2.3.3. App Risk Category

Once an app a is classified by the ML model and its risk score *R*(*a*) is calculated, the app is assigned a risk category, as shown in Table 4. The algorithm uses a set of threshold parameters *t*_1_, *t*_2_, *t*_3_, and *t*_4_. It is described in detail in our prior work [19]. A somewhat similar method was proposed in [62] where apps were assessed based on permission usage and severity of data compromise.

In [19], the values of the threshold parameters were set and tested at 0.25, 0.50, 0.65, and 0.75 on a very small set of installed apps. The experiments in this study showed that with these thresholds, a relatively high number of benign apps were categorized as medium and high risk. This could mislead the MD user either to remove an app that was not harmful, or to ignore the warning and continue to use a truly malicious app. We raised the values of t_2_ and t_4_ and set the threshold values of *t*_1_, *t*_2_, *t*_3_, and *t*_4_ as 0.25, 0.60, 0.65, and 0.80, respectively.

If the reference set of dangerous permissions changes due to changes in the threat landscape, the risk scores of the permissions in the reference set and the risk scores R(*a*) of the apps resident on the MD will need to be recalculated. Similarly, if the classification ML model is retrained or modified, the apps resident on the device will need to be re-evaluated. The threshold parameters may need to be updated as well, to determine the ones performing best with the new data.

### 2.4. Hybrid App Risk Evaluation Method

The hybrid method for app evaluation involves the use of two ensemble ML models:

1. An ML model for the analysis of API calls made by apps at run time. The model analyzes network traffic data to detect malicious activities performed by apps resident on the MD.
2. An ML model for app classification based on static features (permissions and intents) extracted at run time. We used the model described in Section 2.2 for this purpose.

#### 2.4.1. Data Acquisition

A software tool was developed to gather data about network traffic data and about permission and intent requests that were granted at run time. The tool uses the Android virtual private network (VPN) services and tracks API calls to external services. It captures the amount of data sent or received, the duration of the connection, the network protocol used, the IP address and the URL of the destination host, and the permissions and intent requested at the point of making the API calls.

The data were collected by running a set of 4000 APK samples. The set contained 2000 benign and 2000 malicious samples that were chosen randomly from the training dataset described in Section 2.1. The samples were installed on ten AndroidX Emulators (Nexus 5X), with 200 benign and 200 malicious samples hosted by each emulator. Each emulator was configured with 1 GB RAM, 512 MB SD Card, 2 GB internal storage, 1080× 1920HDPI, and 4 multi-core CPUs in a remote Virtual Machine (VM). The VM hardware included an AMD dual Core (processor) i7-8700 CPU@2.00 GHz, 64 GB RAM, and a 1 TB hard disk drive.

For data capture, the apps in each emulator were executed for five hours daily for a period of two days. In addition, the emulators ran unattended for another seven hours per day. This allowed recording of the background network calls made by apps when the emulated devices were idle. A total of 78,285 unique dynamic activity data samples were collected (Table 5).

#### 2.4.2. Feature Selection

The features that represented actual app run-time behavior were extracted from each emulator’s database. They included the set of 39 features (*S_1_, S_2_, … S_39_*) selected by the filter-based IFD method (Table 1) and the set (*C_1_, C_2_, … C_8_*) of eight specific network traffic characteristics of API calls to external services (Table 6).

All API calls that requested an HTTP, HTTPS, TCP, TLS, or DNS connection were considered. These protocols were chosen because typical malware behavior usually involves stealing sensitive information from the device and transmitting it to a remote server.

#### 2.4.3. Ensemble ML Model for Dynamic Analysis

The ensemble ML model for dynamic app activity classification uses the ensemble RF classifier (with bagging). It was chosen based on our prior work [63]. The ML model achieved high-performance results in detecting malicious traffic to a cloud server, using a small set of 11 features. In an MD context, the RF algorithm was used for the dynamic analysis of app network activities in the models proposed in [32,34,38].

In [63], the model was trained on the NSL-KDD dataset; the selected features included the network service, the duration of the connection, and the amount of data sent and received. These four features correspond to features *C_1_, C_2_, C_3_*, and *C*_5_ in Table 6.

**Figure 3:**
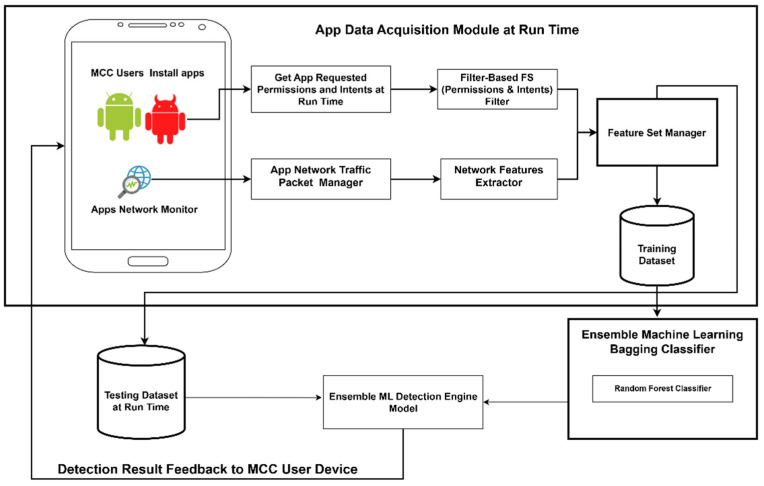
Ensemble ML model for dynamic app activity classification.

The RF classifier used in this study was trained on the experimental dataset of dynamic activity samples using *C_1_, C_2_, C_4_, C_5_, C_7_*, and *C*_8_ as input features. As seen in Figure 3, when the model receives a request for an app activity classification at run time, it analyzes the network traffic data to detect malicious behavior and generate a classification outcome.

## 3. Results

The methods described in Section 2 were deployed in a proof-of-concept prototype of MINDPRES. This section describes the design and implementation of the system including functional and algorithmic description. The results of the prototype performance evaluation were used to assess the effectiveness and the deployment feasibility of MINDPRES.

### 3.1. MINDPRES Prototype Design

**Figure 4:**
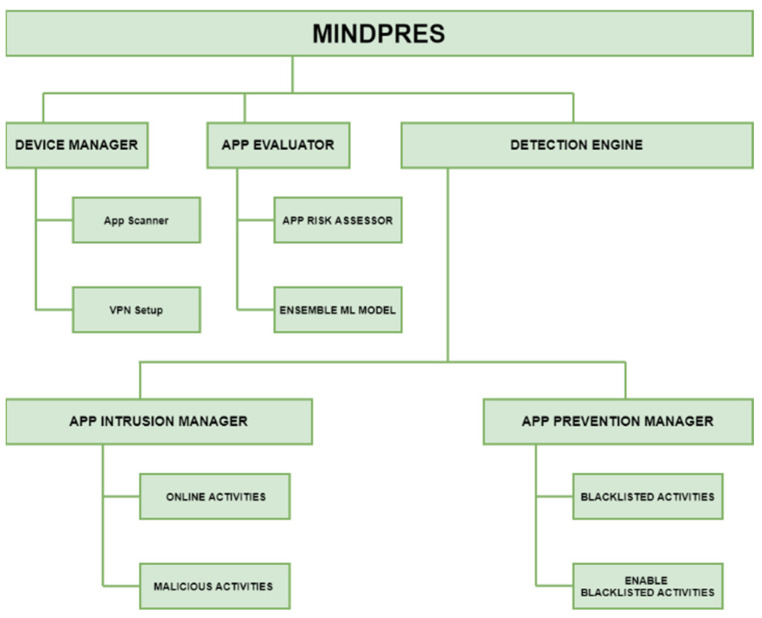
High-level view of MINDPRES prototype system.

The prototype system comprises three sub-systems: a device manager (DM), an app evaluator (AE), and a detection engine (DE) (Figure 4).

#### 3.1.1. Device Manager

**Figure 5:**
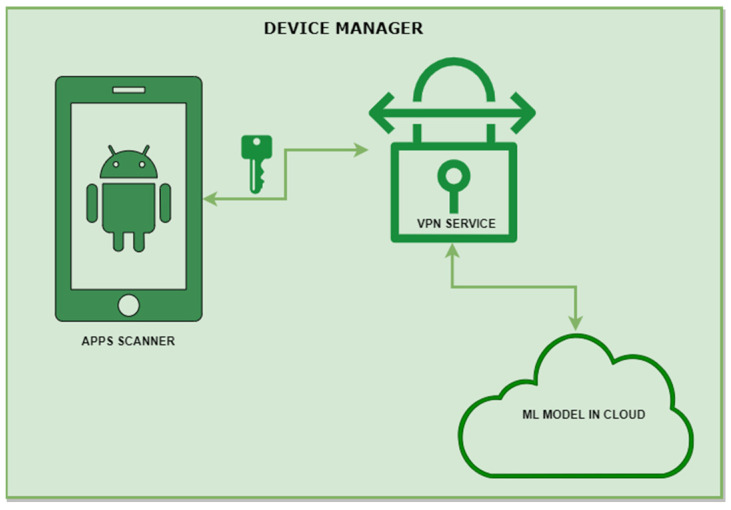
MINDPRES device manager.

Once installed and operational, MINDPRES runs in the background and gathers app network traffic data and run-time permission and intent requests. These are forwarded to the DE component of MINDPRES for further analysis. The DM (Figure 5) is responsible for scanning the apps that reside on the device. It uses the VPN service libraries in the Android OS to prepare the prototype system for monitoring the traffic generated by the resident apps.

The DM does not need root-level access to the OS; however, the device user must grant MINDPRES access to the Android VPN service. The high-level algorithmic design of the DM is shown in Table 7.

#### 3.1.2. App Evaluator

**Figure 6:**
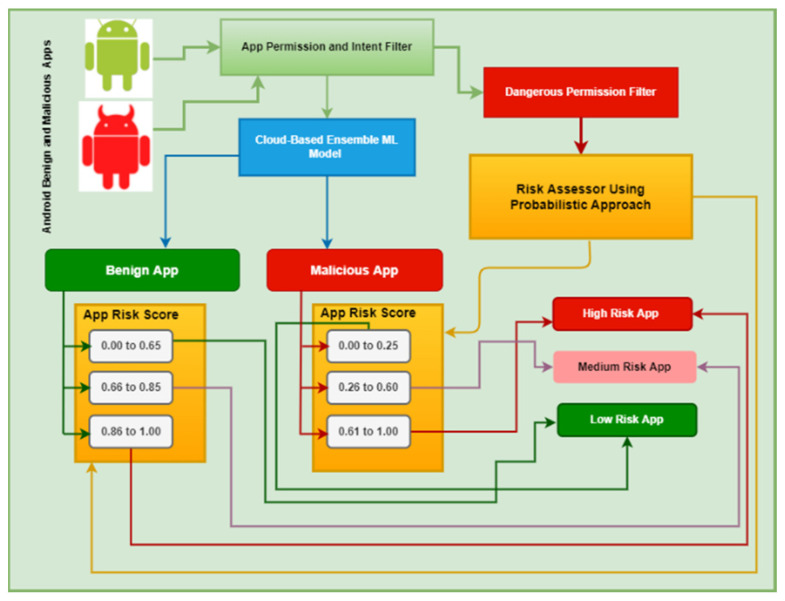
MINDPRES app evaluator.

When MINDPRES is installed and launched for the first time, the AE (Figure 6) assigns a risk category to each user-installed app on the MD (one of high, medium, or low risk). To evaluate an app, the AE reads the content of the manifest file and extracts: (i) the permissions and intents required by the feature selection algorithm of the cloud-based ML model for static analysis described in Section 2.1 and Section 2.2, and (ii) the dangerous permissions used by the app that belong to the reference set of dangerous permissions described in Section 2.3.1. The AE sends a request for classification to the ML model for static analysis.

The ML model returns a benign or malicious classification output for the app under evaluation. Next, the AE computes the risk score value of the app and assigns a risk category using the methods described in Section 2.3.2 and Section 2.3.3. The risk category is sent back to the DM and the MD user is informed.

The same process is followed every time a new app is installed on the MD. The high-level algorithmic design of the AE is shown in Table 8.

#### 3.1.3. Detection Engine

The DE provides the IDPS functionality of MINDPRES. Using the priority list created by the AE during the initial launch of MINDPRES (which is updated every time a new app is installed), the DE monitors app behavior in real time and alerts the user to signs of abnormal activity. Table 9 shows the algorithmic design of the DE.

**Figure 7:**
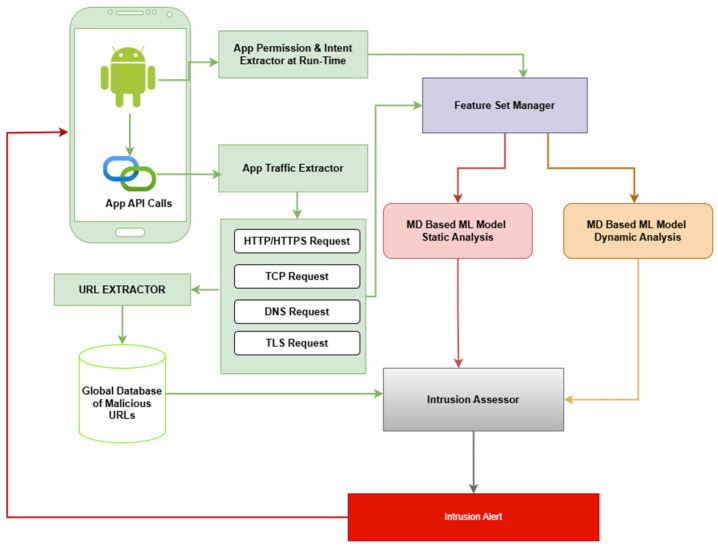
MINDPRES app intrusion manager.

The DE comprises an app intrusion manager and an app prevention manager (Figure 4). The app prevention manager (Figure 7) extracts the permissions and intents granted at run time, selects the required ones, and forwards them to the feature set manager. In this prototype, the DE used is a copy of the model described in Section 2.1 and Section 2.2 for the static analysis of the features gathered at run time.

The app traffic extractor captures the network traffic data required by the ML model for dynamic app evaluation (as described in Section 2.4.2). The app traffic extractor forwards the network traffic data to the feature set manager.

The feature set manager creates the inputs for each of the two ML classification models and sends requests for classification. The responses are sent to the intrusion assessor. In addition, the app traffic extractor checks the URL in the API call against a global database of known malicious URLs. The outcome of the check is also forwarded to the intrusion assessor.

The intrusion assessor raises an intrusion alert if one of the two classification outputs is ‘malicious’ (i.e., either an app has been classified as malicious based on the permissions and intents requested at run time, or the app network behavior has been classified as malicious), or if the check with the malicious URL database returns ‘true’.

The app prevention manager (Figure 4) provides the risk mitigation functionality of MINDPRES. If an intrusion alert is raised for an app, the app prevention manager communicates with the DM and prevents the app from sending data to the destination host. However, the MD user has the option to override the block and allow the connection.

#### 3.1.4. MINDPRES Classes, Interactions and Data Flow

**Figure 8:**
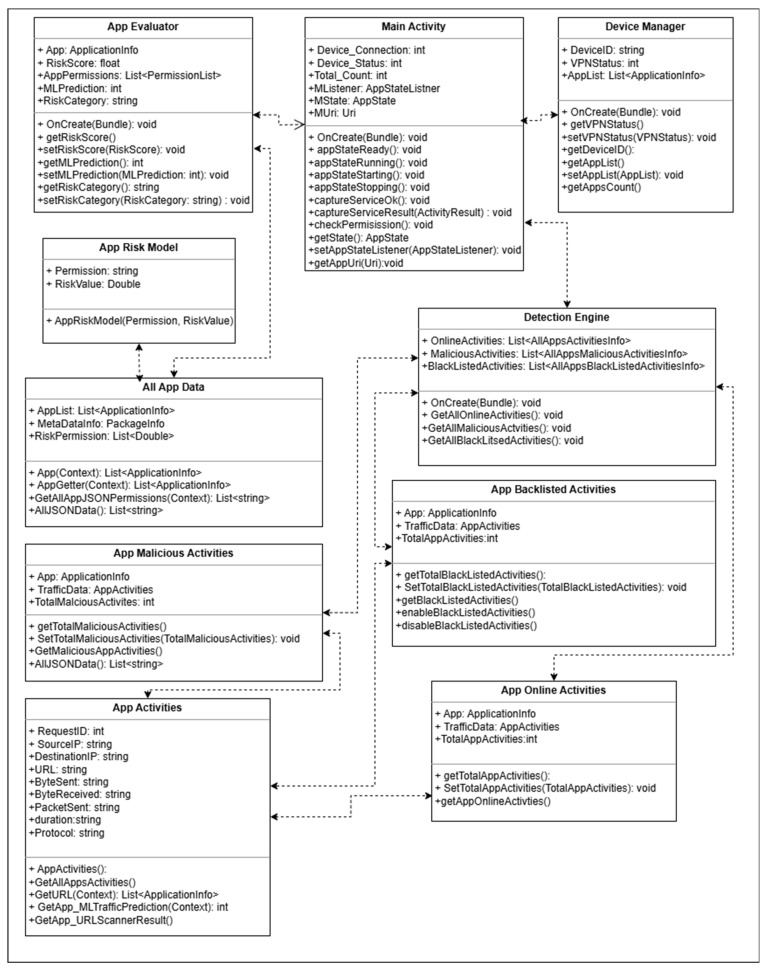
UML class diagram of MINDPRES.

The UML (unified modeling language) class diagram of MINDPRES in Figure 8 shows the classes that make up the prototype system, including the structure of each class and how each class interacts with the other classes. The main activity class is the backbone of the prototype. It has three associated classes that correspond to the system components described above (i.e., the device manager class, the app evaluator class, and the detection engine class). Each class comprises several subclasses that support various MINDPRES functionalities.

**Figure 9:**
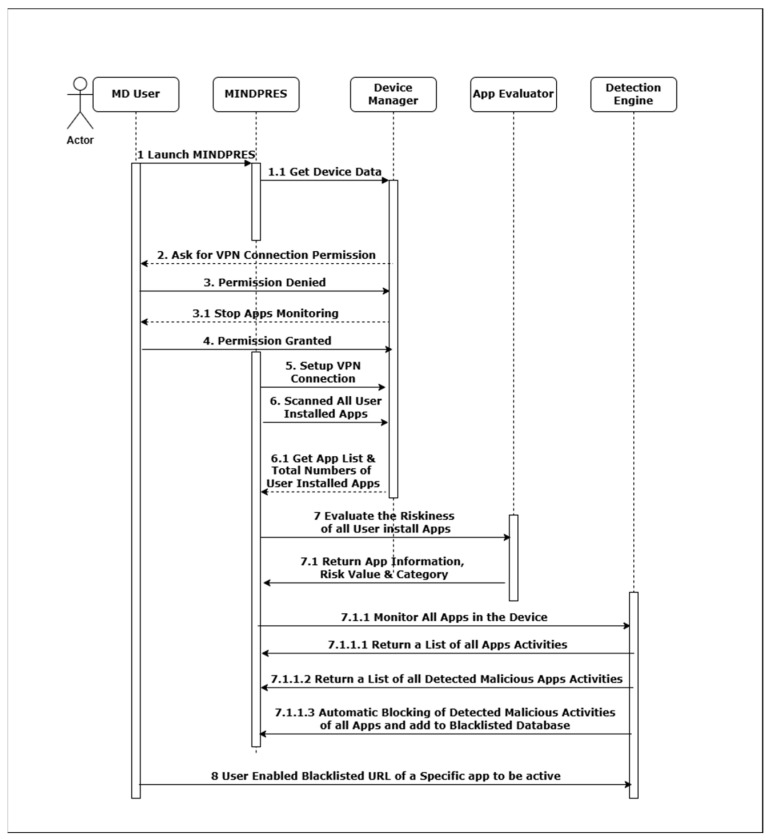
UML sequence diagram of MINDPRES.

**Figure 10:**
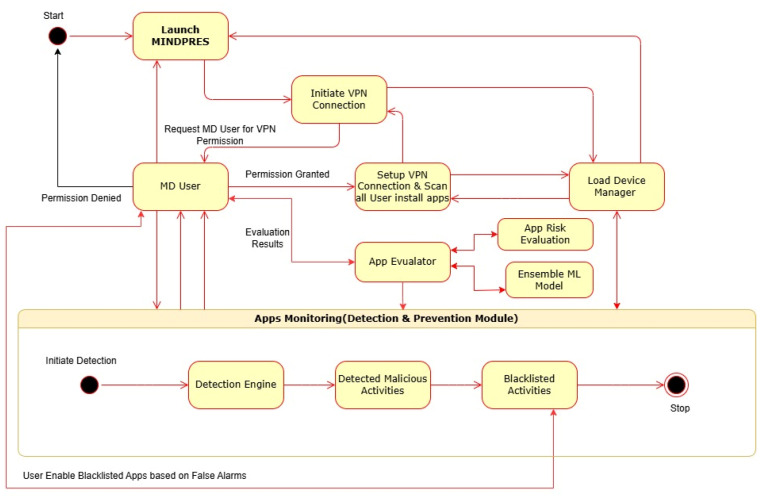
UML activity diagram of MINDPRES.

The interactional activities amongst the MINDPRES objects and the interactions with the MD user are visualized in the UML sequence diagram shown in Figure 9 while the system’s dynamic aspects are visualized in the UML activity diagram in Figure 10. For a more detailed description of the diagrams, see Supplementary Materials, Tables S1–S3.

### 3.2. MINDPRES Prototype Implementation and User Interface

The MINDPRES prototype system includes a MINDPRES mobile app. It contains the DM and the DE components and requires Android 7.0 OS or a higher version. The AE resides on the cloud end of MINDPRES (AWS cloud server). The MINDPRES app communicates with the AWS cloud server using TCP/IP. The MINDPRES app contains copies of the two ensemble ML models that were implemented using Python programming language using the libraries Scikit-learn, Pandas, and NumPy. The libraries have been used in prior research and have proved to be robust and reliable [64].

**Figure 11:**
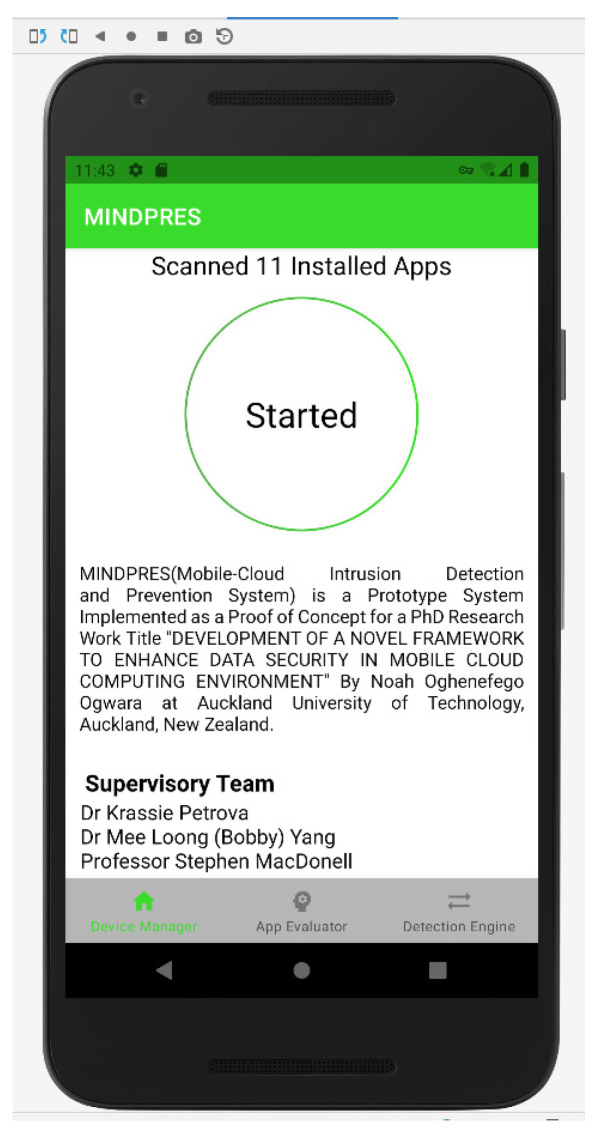
DM user interface.

Once trained, the models were deployed to the AWS cloud container. The device end components of the MINDPRES prototype were developed in Java, using the Android Studio IDE (integrated development environment). Java enabled native access to the device’s low-level resources such as the VPN service and the package manager while the Java libraries provided support for communicating with the Android OS kernel and intercepting apps’ API calls. The extended markup language (XML) in Android Studio was used to design the user interface (UI) of three main functional components of MINDPES (DM, DE, and AE). The DM screen is the first screen the users see when the MINDPRES app is launched. The user is asked to allow access to the VPN service so that MINDPRES can start monitoring the activities of the device-resident apps (Figure 11).

**Figure 12:**
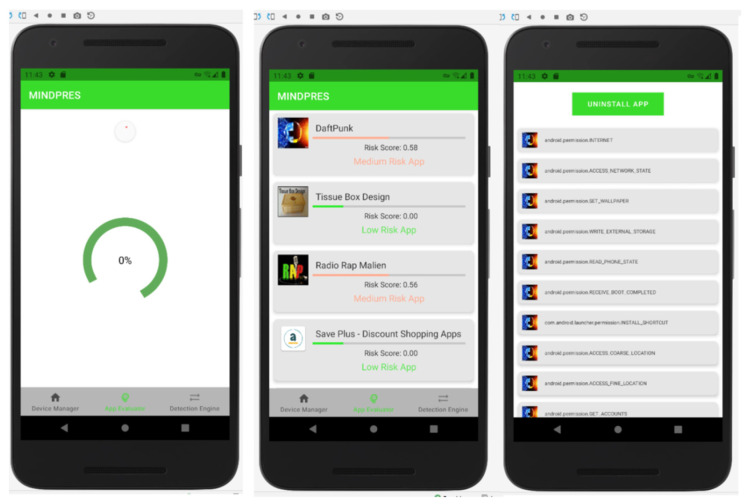
AE user interface.

The AE screen shows the risk score and risk category of each app that has been evaluated. The system displays the permissions requested by each app and provides an option to uninstall apps that may be harmful (Figure 12).

**Figure 13:**
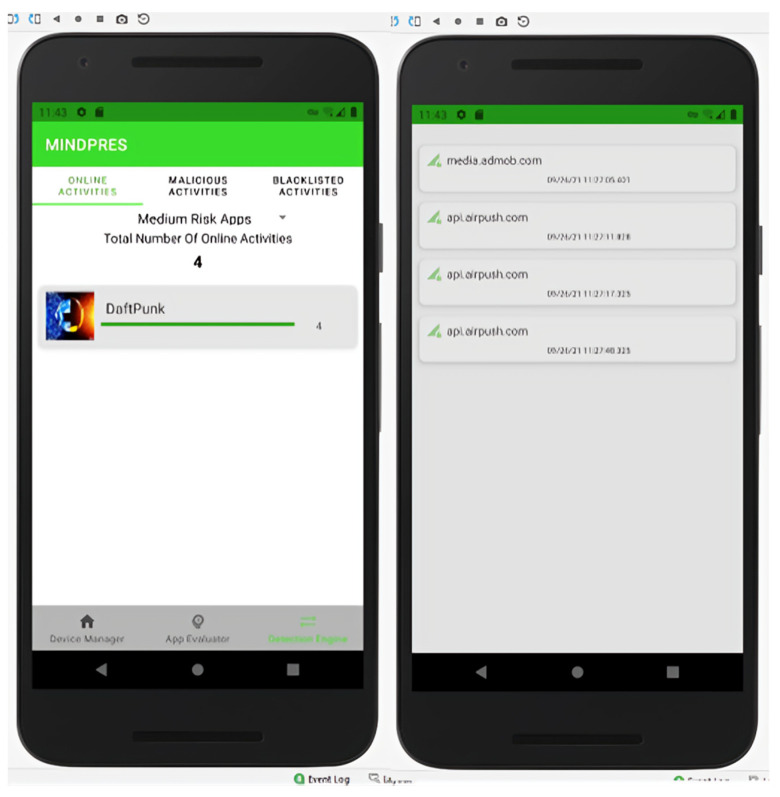
DE user interface.

The DE screen contains several tabs. The MD user can see the online requests made by the apps (including a list of API calls to external URLs and calls’ details), the apps that have made malicious API calls, and URLs flagged as malicious (Figure 13). The MD user may enable or disable a blacklisted URL and a blocked API call. 

### 3.3. MINDPRES Prototype Evaluation

The evaluation assessed the effectiveness of the proposed approach and the feasibility of deploying MINDPRES on MD, including energy consumption. The evaluation followed a staged process, with several real-life experiments conducted. The first stage involved the evaluation of the effectiveness of the static risk analysis method. The second stage involved assessing the effectiveness of the hybrid analysis method.

#### 3.3.1. Experiment Setup

A testbed of 1000 apps was built and used to evaluate MINDPRES performance. It comprised 600 benign and 400 malicious apps. The benign apps were downloaded from the Google Play store in November 2021. The selection included the top 30 most popular apps from each of the 20 app categories available. Only apps with at least one million user downloads were considered. The 400 malicious apps were obtained from the malware dataset CICMalDroid2020 [65], with an equal number of samples from each of the four categories of adware, banking malware, SMS malware, and risk malware). The APK files of the benign and malicious testbed apps were checked using the VirusTotal services [66] to ascertain whether the apps were malicious or not. Applying the criteria used in our prior experiments [19], an app was confirmed as malicious if at least 15 internal antivirus engines in VirusTotal found it malicious. The outcomes did not indicate any misclassification.

The MINDPESS app and the testbed apps were installed on five Android devices labeled A, B, C, D, and E. Table 10 shows the characteristics of the testbed devices and the number of benign and malicious apps installed on each. Table 11 and Table 12 show the distribution of the apps according to category.

#### 3.3.2. Testing the App Evaluator

The first stage focused on the static analysis performed by the AE. The results are presented in Table 13. It shows the number of apps classified as benign and malicious, and the number of apps evaluated as low, medium, and high risk.

Of the 600 benign apps downloaded from the Google Play store, 568 apps (94.67%) were correctly classified as benign. Of the 400 malicious apps, 380 apps (95.00%) were classified correctly as malicious.

In the case of benign apps, 563 apps (93.83%) were evaluated as low risk, 35 apps (5.83%) were evaluated as medium risk, and 2 apps (less than 1%) were evaluated as high risk. About 5% of the benign apps had a zero-risk score. All apps in categories B2, B3, B7, and B8 were determined as low risk; their risk score values were in the range [0.00, 0.60]. The risk score values of the apps in the other app categories were in the range (0.60, 0.80]. Two Google Play benign apps (from B6 and B9) were evaluated as high risk.

In the case of malicious apps, 3 apps (less than 1%) were evaluated as low risk, 294 apps (73.60%) were evaluated as medium risk, and 103 apps (25.75%) were evaluated as high risk. All apps in app categories M1, M2, M4, and M5 were evaluated as either medium or high risk, with risk scores between 0.25 and 1.00. Three apps (from categories M3 and M6) were assigned a low-risk category.

#### 3.3.3. Testing the Detection Engine

Table 14 shows the outcomes of the hybrid analysis using static features extracted at run time and dynamic analysis based on network traffic data.

As the detection engine monitors the actual behavior of the apps both when the user is active and when the device is idle, the evaluation experiment was carried out over a two-week timeframe. During the experiment, each device was used for two hours daily, by running all testbed apps that resided on the device.

As expected, the total number of malicious activities performed by malicious apps (4433) was significantly higher compared to the number of malicious activities performed by benign apps (31). A total number of 371 malicious apps were detected as performing malicious activities, compared to 14 benign apps performing malicious activities. In both app categories and across all devices, a significant number of activities were performed when the device was idle (20.07% of all activities).

#### 3.3.4. Prototype Effectiveness

As seen in Table 13, the ensemble ML model for static analysis effectively distinguished between benign apps and malicious apps, with over 90% of apps in each category classified correctly. On average, 94.80% of the apps were classified correctly; in particular, all apps in categories B1, B2, B3, B7, and B8 were correctly classified. A small number of benign apps (32, or 5.33%) were misclassified, as the APK files of these apps contained requests for permissions and intents commonly used by malicious apps. However, the app evaluator assigned a high-risk category to only two of these apps (one from each of Google Play categories B6 and B9) as they used a significant number of dangerous permissions, and their risk scores were above the high-risk threshold for malicious apps. The rest were assigned a medium-risk category.

Overall, only a small number of benign apps were categorized as medium or high risk. In this, this study has intentionally taken a conservative approach, which is common in security. That is, we would rather flag potentially malicious apps even if they are not so that they can be checked, as opposed to being more permissive and missing some malicious apps. Such apps are potentially dangerous as they can be later manipulated to perform malicious activities by hostile app developers. Therefore, alerting the MD user is appropriate (the app evaluator assessment is only informative, it does not prevent apps from running). The user needs to be warned to be careful when granting permissions to medium-risk apps and treat them with caution due to the possibility of malicious manipulation.

Furthermore, 20 (5.00%) malicious were wrongly classified as benign, as they requested permissions and intents commonly used by benign apps. Subsequently, three of these apps (from the M3 and M6 malware categories) received a low-risk score and were assigned a low-risk category; the MD user would not be alerted to the threat posed by these apps. The rest of the misclassified malicious apps were assigned a medium-risk category, alerting the MD user to the danger.

These results demonstrate the robustness of the static evaluation method deployed in MINDPRES and how calculating the app risk score helps refine the outcome of the static evaluation method. Malicious apps are designed to evade detection, but the app evaluator ‘missed’ only three of them. Still, the number of malicious apps that were assigned only a medium-risk category was relatively high. This may mislead the user to treat medium-risk apps as not dangerous enough and to keep them in use. As the system is used, we will continue to learn from the outcomes and refine the algorithms to increase accuracy and finetune the threshold values used for the categorization of malicious apps.

Table 15 shows the MINDPRES performance evaluation results. The results indicate that the prototype system can detect efficiently intrusion activities caused by malicious apps at the user layer of the MCC environment. Overall, the prototype system achieved a CA of 90% or higher in all devices, for both methods. The static method achieved a better CA than the hybrid method in devices A, C, and E. In devices B and D, the hybrid method outperformed the static one.

The PR values show that the hybrid method performed better in detecting malicious activities compared to the static method, with a PR over 94% for all devices used in the experiment. Similarly, the hybrid method had a better FPR in all devices, with as low as 1.11% for device C. However, the FAR, the recall rate (RC), and ER of the hybrid approach were better than the FAR, RC, and ER of the static approach in only two devices (B and D).

#### 3.3.5. Energy Consumption

**Figure 14:**
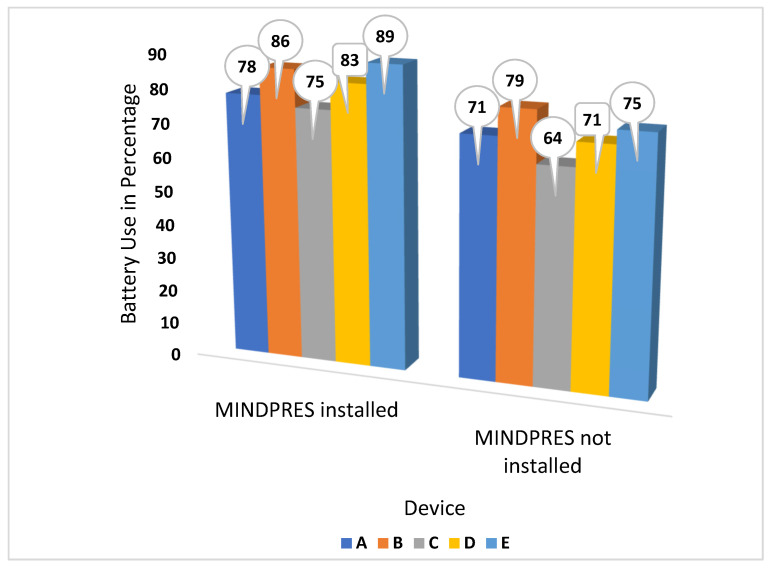
Energy consumption of testbed devices with and without MINDPRES installed.

Typically, MDs are resource-constrained; therefore, it is important to ensure that an IDPS does not overload the device. To gauge the MINDPRES energy consumption, the battery usage of the MDs used in the experiment was recorded both with MINDPRES installed and uninstalled (Figure 14).

Overall, the devices’ energy consumption was only slightly higher when MINDPRES was installed. The energy consumption of device E was very high which may be explained by the type of benign apps installed on the device such as sports and dating. Sports apps have particularly high energy requirements due to the high number of network activities they engage in.

## 4. Discussion

This study explores the use of a combination of app permissions, intents, API calls. URL requests, and network traffic data to analyze static and dynamic app behavior. It applies a hybrid ML-based approach towards malware detection on Android MDs connected to the mobile cloud and proposes MINDPRES, a distributed system in which static analysis is performed at the cloud end and hybrid analysis is performed at the device end.

### 4.1. Comparison with Prior Work

We compared the results of our study with results reported in a selection of related studies on ML-based malware detection systems for Android devices, including static, dynamic, and hybrid approaches. We compared MINDPRES and the other models in terms of features, datasets, sample size, ML algorithms, and the most reported metrics CA, FM, and FPR (Table 16).

As in more recent work [38,39,40,45], MINDPRES deploys a hybrid approach. It performs an analysis of both static and dynamic features as also suggested in [28]. The static features (permissions and intents) and the dynamic features (run-time granted permissions and intents, and network traffic characteristics) have also been used widely. Differently from other proposed solutions, the static method in MIMDPRES considers both the classification output of an ML model and the usage pattern of permissions considered particularly dangerous; in addition, the outcome of the static analysis method is used to prioritize app behavior monitoring and avoid overloading the device. Furthermore, the hybrid analysis method considers both run-time network activities and permission and intent requests.

The ensemble classifiers used in MINDPRES include algorithms that have been well-tested in prior work and have yielded good performance results. Furthermore, the source datasets used in our study have also been used by other researchers, which adds to the credibility of our work. The number of data samples used to train the ML models also compares well to most of the studies, with only two hybrid analysis studies [39,45] and one static analysis study [37] having used significantly larger sets of malicious samples.

All reviewed studies have conducted their experimental work in laboratory conditions while MINDPRES was installed on real-life devices and used to generate the performance evaluation data. The results compare well with other related research. Importantly, the CA of the best-performing MINDPRES hybrid method (97.14%) is well within the range of 95.54% to 99.97% of the CA achieved in the other hybrid studies. Similarly, the CA of the static methods (98.13%) is within the range of 94.11% and 99.40% of the CA of the other static methods. The FPR of the hybrid method (1.11%) is the lowest among all reported FPR values. The FPR of 2.15% of the static method also compares well to the rest of the studies. The FM of the static method (98.55%) is sufficiently high in comparison to other models. However, the FM of the hybrid method (96.08%) is at the lower end of the range of FM values in the other hybrid studies. This can be explained by the relatively high number of false negative (FN) values which decreases the RC value. Training on a larger and more comprehensive dataset may improve the FN value.

Finally, none of the studies has investigated in depth the feasibility of deploying the proposed ML models on real MD. In contrast, we have built and tested a prototype system and have demonstrated the feasibility of the solution.

### 4.2. MINDPRES Advantages

Work on proposing feasible-to-implement systems for the detection of malicious activities executed by apps resident on MDs in the MCC environment is still limited [48,55]. MINDPRES offers several advantages in comparison to the systems proposed in prior work, including most recent studies: (i) Differently from the models reviewed, when working in hybrid mode, MINDPRES extracts both static and dynamic features at run time, including granted permissions and intents. This ensures that malware detection is guided by the actual app behavior rather than by the app manifest file. (ii) The ML model for static risk analysis that is used by both the app evaluator (in the cloud) and by the detection engine (on the device) deploys works effectively with a relatively small feature set. (iii) The dynamic feature extraction process does not require need a specialized safe environment. (iv) MINDPRES does not require app instrumentation, or root-level access to monitor app behavior in real time. Rather, apps are monitored using the device’s VPN service. This makes the proposed system highly implementable. (v) For efficiency, MINDPRES prioritizes monitoring app activities based on the outcomes of the static analyses of the apps installed on the device. (vi) The app risk score calculations that use the risk scores of a reference set of dangerous permissions are performed at the cloud end of MINDPRES. The system can be easily adjusted to accommodate a new set of topmost dangerous permissions, following Google’s updates to the Android OS; (vii) MINDPRES distributes the computational processes between the MD and a cloud server to avoid overloading the MD.

### 4.3. Study Contributions

This study makes several important contributions. First, it proposes a software system for detecting malicious app behavior; the system applies an unobtrusive hybrid analysis approach that takes into consideration MD resource constraints.

Second, the study demonstrates the feasibility of the proposed software system by developing and evaluating a proof-of-concept system prototype of a distributed (just-based and cloud-based) IDPS for the protection of MDs connected to the mobile cloud.

Third, the study validates two innovative methods proposed in our earlier work: (i) the IFD method for selecting static features, and (ii) the stochastic method for calculating app risk scores. Both are used in the static app evaluation performed by the app evaluator.

Fourth, the models and methods developed and used in this study offer a data-driven framework for developing a flexible distributed security protection system for Android MDs. The ML models are trained off-device; they can be easily re-deployed on the participating MDs if modified, including the selection of a new set of features by the IFD. This way, the models can be kept current, and/or rebuilt for better performance. Furthermore, deploying the app evaluator in the cloud allows for a fast and seamless response to changes in the threat environment.

### 4.4. Research Limitations and Concluding Remarks

This research has several two major limitations. First, the proof-of-concept ML model for the analysis of app network activities at run time was trained on a relatively small set of samples collected experimentally. To achieve a better performance, the model can be retrained on a larger dataset. Second, the model was built using one selected classifier. Deploying other classifiers or ensembles including DL algorithms may lead to better performance. Third, the module used a very small set of dynamic features. Further research is needed to determine the best-performing set of dynamic features.

Furthermore, as a distributed system MINDPRES depends on the security of the communication channel and the security of the cloud service. Further research can investigate developing a comprehensive MCC protection system that addresses security issues at the other layers of the MCC infrastructure: the mobile communication channel and MC service provider.

Based on the results obtained in the experiments and the discussion above, it can be concluded that MINDPRES is a feasible solution to the research problem driving this study. The system can be deployed on any Android mobile device connected to an MCC environment. By listening to the permission, intent, URL requests, and API calls that apps make in real time, the system monitors the actual execution of the apps installed on the device and provides an effective defense against threats to the MD and the MMC environment posed by malicious apps.

## Figures and Tables

**Table 1:** Features used by the ML model for static analysis.

Feature ID	Feature Name
*S_1_*	ACCESS_COARSE_LOCATION
*S_2_*	ACCESS_FINE_LOCATION
*S_3_*	ACCESS_LOCATION_EXTRA_COMMANDS
*S_4_*	ACCESS_WIFI_STATE
*S_5_*	BROADCAST_STICKY
*S_6_*	CALL_PHONE
*S_7_*	CHANGE_CONFIGURATION
*S_8_*	CHANGE_NETWORK_STATE
*S_9_*	CHANGE_WIFI_STATE
*S_10_*	DISABLE_KEYGUARD
*S_11_*	GET_ACCOUNTS
*S_12_*	GET_TASKS
*S_13_*	KILL_BACKGROUND_PROCESSES
*S_14_*	MODIFY_AUDIO_SETTINGS
*S_15_*	MOUNT_UNMOUNT_FILESYSTEMS
*S_16_*	READ_CONTACTS
*S_17_*	READ_EXTERNAL_STORAGE
*S_18_*	READ_LOGS
*S_19_*	READ_PHONE_STATE
*S_20_*	READ_SMS
*S_21_*	RECEIVE_BOOT_COMPLETED
*S_22_*	RECEIVE_SMS
*S_23_*	RECORD_AUDIO
*S_24_*	RESTART_PACKAGES
*S_25_*	SET_WALLPAPER
*S_26_*	SYSTEM_ALERT_WINDOW
*S_27_*	VIBRATE
*S_28_*	WAKE_LOCK
*S_29_*	WRITE_EXTERNAL_STORAGE
*S_30_*	WRITE_SETTINGS
*S_31_*	ACTION_BOOT_COMPLETED
*S_32_*	ACTION_PACKAGE_ADDED
*S_33_*	ACTION_PACKAGE_REMOVED
*S_34_*	ACTION_SEARCH
*S_35_*	ACTION_USER_PRESENT
*S_36_*	ACTION_VIEW
*S_37_*	CATEGORY_BROWSABLE
*S_38_*	CATEGORY_DEFAULT
*S_39_*	CATEGORY_HOME

**Table 2:** Performance evaluation results including proposed ensemble classifier (in percentage).

ML Model	TP	FP	TN	FN	CA	ER	PR	FM	FPR	FAR
Ensemble (DT, RF, KNN)	3598	43	1958	63	98.13	1.87	98.82	98.55	2.15	1.93
DT	3421	185	1816	240	92.78	7.22	95.29	94.36	8.45	7.50
RF	3496	174	1827	165	94.00	6.00	95.58	95.35	8.05	6.47
KNN	3484	201	1800	177	93.50	6.50	94.80	94.98	9.55	7.19

**Table 3:** Reference set of dangerous permissions used in this study.

Permission ID	Permission Name
*P_1_*	WRITE_EXTERNAL_TORAGE
*P_2_*	READ_PHONE_STATE
*P_3_*	ACCESS_COARSE_LOCATION
*P_4_*	ACCESS_FINE_LOCATION
*P_5_*	GET_TASKS
*P_6_*	READ_EXTERNAL_STORAGE
*P_7_*	SYSTEM_ALERT_WINDOW
*P_8_*	READ_LOGS
*P_9_*	MOUNT_UNMOUN_FFILESYSTEMS
*P_10_*	CAMERA
*P_11_*	RECORD_AUDIO
*P_12_*	GET_ACCOUNTS
*P_13_*	CALL_PHONE
*P_14_*	WRITE_SETTINGS
*P_15_*	SEND_SMS

**Table 4:** Determining the risk category of an app based on its classification and risk score value.

ML Classification Output for App *a*	R(*a*) Belongs to	Risk Category of App *a*
Malicious	[0, *t*_1_]	Low risk
	(*t*_1_, *t*_3_]	Medium risk
	(*t*_3_, 1]	High risk
Benign	[0, *t*_2_]	Low risk
	(*t*_2_, *t*_4_]	Medium risk
	(*t*_4_, 1]	High risk

Note: 0 < *t*_1_ < *t*_2_ < *t*_3_ < *t*_4_ < 1.

**Table 5:** Dynamic activity data samples.

Emulator	1	2	3	4	5	6	7	8	9	10	Total
Benign Apps	3578	3099	4329	2988	4005	3021	3566	5002	3944	2991	36,523
Malicious Apps	4024	5600	5023	3456	3878	4098	5008	3207	3456	4012	41,762
Total recorded	7602	8699	9352	6444	7883	7119	8574	8209	7400	7003	78,285

**Table 6:** Network traffic characteristics.

ID	Type	Description
*C_1_*	Protocol	The network communication protocol requested
*C_2_*	Duration	The duration of the connection between the MD and the destination host
*C_3_*	Domain URL	The URL of the destination host servicing the API call
*C_4_*	Packets Sent	The number of packets sent (by the MD)
*C_5_*	Packets Received	The number of packets received (from the destination host)
*C_6_*	Destination IP	The IP address of the destination host
*C_7_*	Source Bytes	The amount of data sent by (by the MD)
*C_8_*	Destination Bytes	The amount of data received (from the destination host)

**Table 7:** High-level algorithmic description of device manager.

Steps	Description
Step 1	*Let **VPNStatus = 0** and Initialize VPN Service Connection and Prompt User for permission to monitor all apps*
Step 2	*IF VPN Service Connection is granted by the user, Then Go to Step 3 Otherwise Go to Step*
Step 3	*Setup VPN Service Connection for the Device and Set **VPNStatus** = 1 (Ready)*
Step 4	*Scanned all Apps that are Installed by the User and Set Array **DefaultAppList** to AllUserInstallApps*
Step 5	*Let **AppsCount** = number of records in array **DefaultAppList and** Set **TotalUserInstallApps = AppsCount***
Step 6	*Stop VPN Service Connection and Set **VPNStatus**=**0***
Step 7	*End of IF structure in Step 2*
Step 8	***OUTPUT*** *: **VPNStatus, TotalUserInstallApps***
Step 9	*Exit*

**Table 8:** High-level algorithmic description of app evaluator.

Steps	Description
INPUT	***DangerousPermissionList*** *, **EnsemblePermissionIntentList, PermissionRiskValue***
Step 1	*For Each App X in **DefaultAppList** repeat step 2, 3, 4, 5, and 6*
Step 2	Extract the Permission and Intent demanded by app X contained in the Features Listed in **EnsemblePermissionIntentList** as **appPermissionIntentLis**t and Set array **PI** = **appPermissionIntentLis**t
Step 3	Extract the dangerous Permission demanded by app X contained in the Features Listed in **DangerousPermissionList** as **appDangerousPermissionLis**t and Set array **DP** = **appDangerousPermissionLis**t
Step 4	Compute the **RiskScore** of app X with the selected dangerous permission in **DP** and Extract the Permission Risk Value for each permission contained in **PermissionRiskValue** that exists in **DP**
Step 5	Get the result of the ensemble ML prediction for app X using the features extracted from Step 2 in array **PI** and store the result return in a variable **MLResult** as integer (0 is benign and 1 is malicious)
Step 6	*IF **RiskScore** in Step 4 is greater than or equal to 0.75 and **MLResult = 0** Then Set **RiskCategory** to **“High Risk App”** and go to Step 12 **Otherwise** go to step 7*
Step 7	*IF **RiskScore** in Step 4 is greater than or equal to 0.50 and **MLResult = 0** Then Set **RiskCategory** to **“Medium Risk App”** and go to Step 12 **Otherwise** go to step 8*
Step 8	*IF **RiskScore** in Step 4 is greater than or equal to 0.00 and **MLResult = 0** Then Set **RiskCategory** to **“Low Risk App”** and go to Step 12 **Otherwise** go to step 9*
Step 9	*IF **RiskScore** in Step 4 is greater than or equal to 0.65 and **MLResult = 1** Then Set **RiskCategory** to **“High Risk App”** and go to Step 12 **Otherwise** go to step 10*
Step 10	*IF **RiskScore** in Step 4 is greater than or equal to 0.25 and **MLResult = 1** Then Set **RiskCategory** to **“Medium Risk App”** and go to Step 12 **Otherwise** go to step 11*
Step 11	*Set **RiskCategory** to **“Low Risk App”** and go to Step 12* ***End of If Structure in Step 6***
Step 12	**OUTPUT *RiskScore, RiskCategory***
Step 13	*End of Step 1 For -Loop*
Step 14	*Exit*

**Table 9:** High-level algorithmic design of detection engine.

Steps	Description
Var	Array<string>: **AppsActivities, MaliciousAppsTraffic, BlackListedAppsTraffic**
INPUT	***VPNStatus*** *, **EnsemblePermissionIntentList, TrafficDataList, DefaultAppTrafficList***
Step 1	*IF **VPNStatus** = 1 Then Go to Step 2 Otherwise Go to Step 16*
Step 2	*For Each App X in **DefaultAppList** repeat step 3, 13, and 14*
Step 3	*For Each Traffic Data of App X in **DefaultAppTrafficList** repeat step 4, 5, 6, 7, 8, 9, and 10*
Step 4	Extract the Permissions and Intents demanded by app X at run-time whenever an online Request is made contained in the Features Listed in ***EnsemblePermissionIntentList*** as ***appPermissionIntentLis****t* and Set array ***PI*** = ***appPermissionIntentLis****t*
Step 5	Extract the Traffic Network Data by app X contained in the Features Listed in ***TrafficDataList*** as ***appTrafficData*** and Set array **TD** = ***appTrafficData*** *and add Traffic data for app x in array* **AppsActivities**
Step 6	Extract the URL call by app X contained in the ***TrafficDataList*** as ***appTrafficURL*** and Set array **TURL** = ***appTrafficURL***
Step 7	Construct App network traffic dataset from **TD** in step 5 and **PI** in step 4 and set **AppTrafficMLData** as the new dataset for each traffic request consisting of API calls, Permissions, and Intent
Step 8	Get the result of the ensemble ML prediction for app X using the features constructed from Step 7 in array **AppTrafficMLData** and store the result return in a variable ***MLTrafficResult*** as integer (0 is benign and 1 is malicious)
Step 9	Get the result of the Malicious Global Database scanner for app X using the URL extracted from Step 6 in array **TURL** and store the result return in a variable ***URLTrafficResult*** as integer (0 is benign and 1 is malicious)
Step 10	*IF **MLTrafficResult** = 1 OR **URLTrafficResult*** ***= 1*** *Then **ADD** the traffic data in* **TD** *from step 5 for app X to* **MaliciousAppsTraffic** *and also automatically block **TD** and **ADD** to* **BlackListedAppsTraffic** for app X and *go to Step 11 **Otherwise** go to step 12*
Step 11	***End of If Structure in Step 10***
Step 12	End of Inner For-Loop in Step 3
Step 13	Set **TotalAppActivites** = Total Number of Records in **AppsActivities** in Step 5 Set **TotalMaliciousAppActivites** = Total Number of Records in **MaliciousAppsTraffic** in Step 10 Set **TotalBlacklistedAppActivites** = Total Number of Records in **BlackListedAppsTraffic** in Step 10
Step 14	**OUTPUT: TotalAppActivites, TotalMaliciousAppActivites, TotalBlacklistedAppActivites, AppsActivities, MaliciousAppsTraffic, BlackListedAppsTraffic** for app X
Step 15	*End of Outer For -Loop **in** Step 2*
Step 16	*Exit*

**Table 10:** Experiment setup.

Device ID	Device Characteristics	Number of Benign Apps Installed	Number of Malicious Apps Installed
A	EE Tablet HTC Nexus 9.8.9, 1.8 GB RAM, 32 GB Internal storage (High Tech Computer Corporation), TaoYan City, Taiwan	240	200
B, C, D, E	Samsung Galaxy Tab A (SM-T380), 2 GB RAM, 16 GB Internal storage, Samsung Electronics Co., Ltd., Suwon-Si, Republic of Korea	90	50

**Table 11:** Benign apps from Google Play installed on each device.

Category ID	Category Name	Device
B1	Smartwatch	A
B2	Art & Design	A
B3	Beauty	A
B4	Business	A
B5	Communication	A
B6	Education	A
B7	Events	A
B8	Food and Drink	A
B9	Shopping	B
B10	Social	B
B11	News & Magazines	B
B12	Finance	C
B13	Entertainment	C
B14	Lifestyle	C
B15	Music & Audio	D
B16	Maps & Navigation	D
B17	Travel and Local	D
B18	Tools	E
B19	Sports	E
B20	Dating	E

Note: A total of 30 apps from each category were included.

**Table 12:** Malicious apps from CICMalDroid2020 installed on each device.

Category ID	Category Name	Number of Apps	Device
M1	Adware	100	A
M2	Banking Malware	100	A
M3	SMS Malware	50	B
M4	SMS Malware	50	C
M5	Mobile Riskware	50	D
M6	Mobile Riskware	50	E

**Table 13:** App classification and risk evaluation results.

Testbed Apps	Classification Results		Risk Evaluation Results		
	Benign	Malicious	Low Risk	Medium Risk	High Risk
B1 (Smartwatch)	30	0	29	1	0
B2 (Art and Design)	30	0	30	0	0
B3 (Beauty)	30	0	30	0	0
B4 (Business)	28	2	28	2	0
B5 (Communication)	27	3	27	3	0
B6 (Education)	28	2	28	1	1
B7 (Events)	30	0	30	0	0
B8 (Food and Drink)	30	0	30	0	0
B9 (Shopping)	26	4	26	3	1
B10 (Social)	25	5	25	5	0
B11 (News & Magazines)	28	2	26	4	0
B12 (Finance)	29	1	29	1	0
B13 (Entertainment)	29	1	29	1	0
B14 (Lifestyle)	29	1	28	2	0
B15 (Music & Audio)	27	3	27	3	0
B16 (Maps & Navigation)	28	2	28	2	0
B17 (Travel and Local)	29	1	29	1	0
B18 (Tools)	28	2	28	2	0
B19 (Sports)	28	2	27	3	0
B20 (Dating)	29	1	29	1	0
M1 (Adware)	7	93	0	73	27
M2 (Banking Malware)	3	97	0	85	15
M3 (SMS Malware)	4	46	2	35	13
M4 (SMS Malware)	2	48	0	30	20
M5 (Mobile Riskware)	3	47	0	38	12
M6 (Mobile Riskware)	1	49	1	33	16

**Table 14:** Network activities captured by detection engine.

Device	Actual App Type	Activities When Device in Use	Activities When Device Idle	Number of Malicious Activities	Number of Apps Performing Malicious Activities
A	Benign	1978	597	13	6
B	Benign	899	215	5	2
C	Benign	768	198	2	1
D	Benign	987	231	7	3
E	Benign	1204	149	4	2
A	Malicious	2876	459	1781	187
B	Malicious	768	202	571	48
C	Malicious	1377	599	650	45
D	Malicious	1422	231	679	49
E	Malicious	1009	456	752	42

**Table 15:** MINDPRES performance data.

Device	Analysis Method	TP	FP	TN	FN	CA	ER	PR	RC	FM	FPR	FNR	FAR
A	Static	190	7	233	10	96.14	3.86	96.45	95.00	95.72	2.92	5.00	3.96
	Hybrid	187	6	234	13	95.68	4.32	96.89	93.50	95.17	2.50	6.50	4.50
B	Static	46	4	86	4	94.29	5.71	92.00	92.00	92.00	4.44	8.00	6.22
	Hybrid	48	2	88	2	97.14	2.86	96.00	96.00	96.00	2.22	4.00	3.11
C	Static	48	3	87	2	96.43	3.57	94.12	96.00	95.05	3.33	4.00	3.67
	Hybrid	45	1	89	5	95.71	4.29	97.83	90.00	93.75	1.11	10.00	5.56
D	Static	47	6	84	3	93.57	6.43	88.68	94.00	91.26	6.67	6.00	6.33
	Hybrid	49	3	87	1	97.14	2.86	94.23	98.00	96.08	3.33	2.00	2.67
E	Static	49	5	85	1	95.71	4.29	90.74	98.00	94.23	5.56	2.00	3.78
	Hybrid	42	2	88	8	92.86	7.14	95.45	84.00	89.36	2.22	16.00	9.11

**Table 16:** Comparison with prior work.

Source and Approach	Feature Types	Datasets	Benign App Sample Size	Malicious App Sample Size	ML Algorithm	Best Performance Results
This study: Static	Permissions, Intents	AndroZoo, RmvDroid	9879	18,427	Ensemble (DT, RF, KNN)	CA = 98.13% FM = 98.55% FPR = 2.15%
This study: Hybrid	Permissions, Intents, API calls, Network traffic	AndroZoo, RmvDroid, CicMalDroid2020, Google Play	9879 + 600	18,427 + 400	Ensemble (DT, RF, KNN), RF	CA = 97.14% FM = 96.08% FPR = 1.11%
[22]: Dynamic	Device resource	Google Play	6000	6000	DT	CA = 99.80%
[23]: Dynamic	System calls	Baidu Mobile Application, Market, VirusShare	Not Reported	Not Reported	MCA	CA = 97.85%. PR = 98.70%, FPR = 4.21%
[34]: Dynamic	Permissions, API calls	AndroZoo	14,172	13,719	RF	FM = 94.3%
[38]: Hybrid	Permissions, Intents, Network traffic, Information leakage, Cryptography use	Drebin, Google Play	2500	2500	RF, NB, SVM, DT, K*	FM = 97%
[17]: Static	Permissions	AndroZoo, VirusShare	1959	2113	RF	CA = 94.11%, FM = 93.00%, FPR = 0.00%
[36]: Static	Permissions, API calls, Contextual features	CicMalDroid2020	4100	12,800	RF, LTR, SVM, KNN, DT	CA = 99.4%
[39]: Hybrid	Permissions, Action repetition	Palo Alto Networks Dataset	90,876	104,747	GB, XGB, DT, RF	CA = 99.8% FM = 99.8%
[32]: Dynamic	Network traffic, System calls, Binder calls	CICMalDroid2020	1795	9803	SVM, NB, LLR, DT, RF	CA = 98.08%
[33]: Dynamic	Network traffic, System calls, Binder calls.	CICAndMal2017, SICMalDroid2020	Not reported	Not reported	Ensemble (KNN, SVM, LR, DT)	FM = 97.57%
[40]: Hybrid	API calls, Network traffic, System calls	Android Malware Detection Dataset	9045	3233	SVR	CA = 95.74% FM = 96.38%
[44]: Hybrid	Permissions, Intents, System calls, Contextual features	Drebin, AndroZoo, SICMalDroid2020	16,037	17,069	DNN, SVM, RF, KNN	CA = 99.97% FM = 99.97%
[35]: Static	Permissions, System calls	28 Standard Android Botnet Dataset	3628	3572	DT, RF, ET, LGBM	CA = 98.05%
[37]: Static	Permissions, API calls, App ratings, App downloads	Google Play, Softonic, Android Authority, CNET, Sanddroid	70,000	70,000	Nine ML algorithms and three ML ensemble classifiers, a neural network	CA = 98.80%
[45]: Hybrid	Permissions, API calls, Network traffic, System calls	CICAndMal2017	298,087	104,747	GBT, RC	CA = 93.50%

Notes: ET: Extra Tree; LLR: Logical Regression; LTR: Logistic Regression; K*: K-Star; MCA: Monte Carlo Algorithm; DNN: Deep Neural Network; GB: Gradient Boosting; XGB: XGBoost; LGBM: Light Gradient Boosting Machine; RC: Ridge Classifier; GBT: Gradient Boosted Tree.

## Data Availability

This study used data from datasets and repositories that are publicly available and referenced in the text. The data gathered experimentally may be available upon request.

## Supplementary Materials

The following supporting information can be downloaded at: https://www.mdpi.com/article/10.3390/s25030670/s1, Table S1: Class diagram description; Table S2: Sequence diagram description; Table S3: Activity diagram description.

## References

[1] Sharma M., Kaul A.A. (2024). A review of detecting malware in android devices based on machine learning techniques. Expert Syst..

[2] Gaber M.G., Ahmed M.H. (2024). Malware detection with artificial intelligence: A systematic literature review. ACM Comput. Surv..

[3] Noor T.H., Zeadally S., Alfazi A., Sheng Q.Z. (2018). Mobile cloud computing: Challenges and future research directions. J. Netw. Comput. Appl..

[4] Dey S., Ye Q., Sampalli S. (2019). A machine learning-based intrusion detection scheme for data fusion in mobile clouds involving heterogeneous client networks. Inf. Fusion.

[5] Cinar A.C., Kara T.B. (2023). The current state and future of mobile security in the light of the recent mobile security threat reports. Multimed. Tools Appl..

[6] Wenhua Z., Hasan M.K., Ismail A.F., Yanke Z., Razzaque M.A., Islam S., Budati A.K. (2023). Data security in smart devices: Advancement, constraints and future recommendations. IET Netw..

[7] Palma C., Ferreira A., Figueiredo M. (2024). Explainable machine learning for malware detection on Android applications. Information.

[8] Bostani H., Moonsamy V. (2024). Evadedroid: A practical evasion attack on machine learning for black-box android malware detection. Comput. Secur..

[9] Butt U.A., Amin R., Mehmood M., Aldabbas H., Alharbi M.T., Albaqami N. (2023). Cloud security threats and solutions: A survey. Wirel. Pers. Commun..

[10] Chauhan M., Shiaeles S. (2023). An analysis of cloud security frameworks, problems and proposed solutions. Network.

[11] Nisha O.J., Bhanu S.M.S. (2020). Detection of malware applications using social spider algorithm in the mobile cloud computing environment. Int. J. Ad Hoc Ubiquitous Comput..

[12] Mollah M.B., Azad M.A.K., Vasilakos A. (2017). Security and privacy challenges in mobile cloud computing: Survey and way ahead. J. Netw. Comput. Appl..

[13] Gaurav A., Gupta B.B., Panigrahi P.K. (2023). A comprehensive survey on machine learning approaches for malware detection in IoTt-based enterprise information system. Enterp. Inf. Syst..

[14] Alsmadi A.A., Shuhaiber A., Alhawamdeh L.N., Alghazzawi R., Al-Okaily M. (2022). Twenty years of mobile banking services development and sustainability: A bibliometric analysis overview (2000–2020). Sustainability.

[15] Saranya A., Naresh R. (2023). Efficient mobile security for e-health care application in cloud for secure payment using key distribution. Neural Process. Lett..

[16] Naveed Q.N., Choudhary H., Ahmad N., Alqahtani J., Qahmash A.I. (2023). Mobile learning in higher education: A systematic literature review. Sustainability.

[17] Jannath N.O.S., Bhanu M.S.S. (2021). Detection of malicious Android applications using ontology-based intelligent model in mobile cloud environment. J. Inf. Secur. Appl..

[18] Ogwara N.O., Petrova K., Yang M.L.B., MacDonell S.G., Daimi K., Arabnia H.K., Deligannidis L., Huang M.-S., Tinnetti F.G. (2021). Enhancing data security in the user layer of mobile cloud computing environment: A novel approach. Advances in Security, Networks, and Internet of Things: Proceedings from SAM’20, ICWN’20, ICOMP’20, and ESCS’20.

[19] Ogwara N.O., Petrova K., Yang M.L., MacDonell S.G. (2024). A risk assessment framework for mobile apps in mobile cloud computing environments. Future Internet.

[20] Kumar R., Goyal R. (2019). On cloud security requirements, threats, vulnerabilities, and countermeasures: A survey. Comput. Sci. Rev..

[21] AlAhmad A.S., Kahtan H., Alzoubi Y.I., Ali O., Jaradat A. (2021). Mobile cloud computing models security issues: A systematic review. J. Netw. Comput. Appl..

[22] Ribeiro J., Saghezchi F.B., Mantas G., Rodriguez J., Shepherd S.J., Abd-Alhameed R.A. (2019). An autonomous host-based intrusion detection system for Android mobile devices. Mob. Netw. Appl..

[23] Zhou Q., Feng F., Shen Z., Zhou R., Hsieh M.Y., Li K.C. (2019). A novel approach for mobile malware classification and detection in Android systems. Multimed. Tools Appl..

[24] John T.S., Thomas T., Emmanuel S. (2024). Detection of evasive Android malware using EigenGCN. J. Inf. Secur. Appl..

[25] Verderame L., Ruggia A., Merlo A. (2023). PARIOT: Anti-repackaging for IoT firmware integrity. J. Netw. Comput. Appl..

[26] Casolare R., Fagnano S., Iadarola G., Martinelli F., Mercaldo F., Santone A. (2024). Picker blinder: A framework for automatic injection of malicious inter-app communication. J. Comput. Virol. Hacking Tech..

[27] Shamshirband S., Fathi M., Chronopoulos A.T., Montieri A., Palumbo F., Pescapè A. (2020). Computational intelligence intrusion detection techniques in mobile cloud computing environments: Review, taxonomy, and open research issues. J. Inf. Secur. Appl..

[28] Makhlouf A.M., Ghribi S., Zarai F. (2023). Layer-based cooperation for intrusion detection in mobile cloud environment. Int. J. Mob. Commun..

[29] Sathupadi K. (2023). A hybrid deep learning framework combining on-device and cloud-based processing for cybersecurity in mobile cloud environments. Int. J. Inf. Cybersecur..

[30] Mishra P., Jain T., Aggarwal P., Paul G., Gupta B.B., Attar R.W., Gaurav A. (2024). Cloudintellmal: An advanced cloud-based intelligent malware detection framework to analyze Android applications. Comput. Electr. Eng..

[31] Dahiya A., Singh S., Shrivastava G. (2023). Android malware analysis and detection: A systematic review. Expert Syst..

[32] Bhat P., Behal S., Dutta K. (2023). A system call-based Android malware detection approach with homogeneous & heterogeneous ensemble machine learning. Comput. Secur..

[33] Roy S., Bhanja S., Das A. (2023). AndyWar: An intelligent android malware detection using machine learning. Innov. Syst. Softw. Eng..

[34] Alazab M., Alazab M., Shalaginov A., Mesleh A., Awajan A. (2020). Intelligent mobile malware detection using permission requests and API calls. Future Gener. Comput. Syst..

[35] Kirubavathi G., Anne W.R. (2024). Behavioral based detection of android ransomware using machine learning techniques. Int. J. Syst. Assur. Eng. Manag..

[36] Aljarrah M.N., Yaseen Q.M., Mustafa A.M. (2022). A context-aware android malware detection approach using machine learning. Information.

[37] Mahindru A., Arora H., Kumar A., Gupta S.K., Mahajan S., Kadry S., Kim J. (2024). PermDroid a framework developed using proposed feature selection approach and machine learning techniques for android malware detection. Sci. Rep..

[38] Maryam A., Ahmed U., Aleem M., Lin J.C.-W., Arshad Islam M., Iqbal M.A. (2020). cHybriDroid: A machine learning-based hybrid technique for securing the edge computing. Secur. Commun. Netw..

[39] Shatnawi A.S., Jaradat A., Yaseen T.B., Taqieddin E., Al-Ayyoub M., Mustafa D. (2022). An android malware detection leveraging machine learning. Wirel. Commun. Mob. Comput..

[40] Aldhafferi N. (2024). Android malware detection using support vector regression for dynamic feature analysis. Information.

[41] Miltenberger M., Arzt S. Extensible and Scalable Architecture for Hybrid analysis. Proceedings of the 12th ACM SIGPLAN International Workshop on the State of the Art in Program Analysis.

[42] Surendran R., Thomas T., Emmanuel S. (2020). A TAN based hybrid model for Android malware detection. J. Inf. Secur. Appl..

[43] Obidiagha C.C., Rahouti M., Hayajneh T. (2024). DeepImageDroid: A hybrid framework leveraging visuals transformers and convolutional neural networks for robust Android malware detection. IEEE Access.

[44] Asmitha K., Vinod P., KA R.R., Raveendran N., Conti M. (2024). Android malware defense through a hybrid multi-modal approach. J. Netw. Comput. Appl..

[45] Sonya A., Deepak R.R. (2024). Android malware detection and classification using machine learning algorithm. Int. J. Commun. Netw. Inf. Secur..

[46] Darabian H., Homayounoot S., Dehghantanha A., Hashemi S., Karimipour H., Parizi R.M., Choo K.-K.R. (2020). Detecting cryptomining malware: A deep learning approach for static and dynamic analysis. J. Grid Comput..

[47] Alzaylaee M.K., Yerima S.Y., Sezer S. (2020). DL-Droid: Deep learning based android malware detection using real devices. Comput. Secur..

[48] Yunmar R.A., Kusumawardani S.S., Mohsen F. (2024). Hybrid Android malware detection: A Review of heuristic-based approach. IEEE Access.

[49] Bilot T., El Madhoun N., Al Agha K., Zouaoui A. (2024). A survey on malware detection with graph representation learning. ACM Comput. Surv..

[50] Feng J., Shen L., Chen Z., Lei Y., Li H. (2025). HGDetector: A hybrid Android malware detection method using network traffic and Function call graph. Alex. Eng. J..

[51] Wu Y., Shi J., Wang P., Zeng D., Sun C. (2023). DeepCatra: Learning flow-and graph-based behaviours for Android malware detection. IET Inf. Secur..

[52] Gu J., Zhu H., Han Z., Li X., Zhao J. (2024). GSEDroid: GNN-based android malware detection framework using lightweight semantic embedding. Comput. Secur..

[53] Li T., Luo Y., Wan X., Li Q., Liu Q., Wang R., Jia C., Xiao Y. (2024). A malware detection model based on imbalanced heterogeneous graph embeddings. Expert Syst. Appl..

[54] Li T., Shou P., Wan X., Li Q., Wang R., Jia C., Xiao Y. (2024). A fast malware detection model based on heterogeneous graph similarity search. Comput. Netw..

[55] Sharma Y., Arora A. (2024). A comprehensive review on permissions-based Android malware detection. Int. J. Inf. Secur..

[56] Ogwara N.O., Petrova K., Yang M.L.B. MOBDroid2: An Improved Feature Selection Method for Detecting Malicious Applications in a Mobile Cloud Computing Environment. Proceedings of the 2021 International Conference on Computational Science and Computational Intelligence, Location of Conference.

[57] Allix K., Bissyandé T.F., Klein J., Le Traon Y. AndroZoo: Collecting Millions of Android Apps for the Research Community. Proceedings of the 2016 IEEE/ACM 13th Working Conference on Mining Software Repositories (MSR).

[58] Wang H., Si J., Li H., Guo Y. RmvDroid: Towards a Reliable Android Malware Dataset with App Metadata. Proceedings of the 2019 IEEE/ACM 16th International Conference on Mining Software Repositories.

[59] AlOmari H., Yaseen Q.M., Al-Betar M.A. (2023). A comparative analysis of machine learning algorithms for Android malware detection. Procedia Comput. Sci..

[60] Akhtar M.S., Feng T. (2023). Evaluation of machine learning algorithms for malware detection. Sensors.

[61] Mat S.R.T., Ab Razak M.F., Kahar M.N.M., Arif J.M., Firdaus A. (2022). A Bayesian probability model for Android malware detection. ICT Express.

[62] Yang A., Ma Z., Zhang C., Han Y., Hu Z., Zhang W., Huang X., Wu Y. (2023). Review on application progress of federated learning model and security hazard protection. Digit. Commun. Netw..

[63] Ogwara N.O., Petrova K., Yang M.L. (2022). Towards the development of a cloud computing intrusion detection framework using an ensemble hybrid feature selection approach. J. Comput. Netw. Commun..

[64] Gupta P., Bagchi A. (2024). Essentials of Python for Artificial Intelligence and Machine Learning.

[65] Mahdavifar S., Kadir A.F.A., Fatemi R., Alhadidi D., Ghorbani A.A. Dynamic Android Malware Category Classification using Semi-Supervised Deep Learning. Proceedings of the 2020 IEEE Intl Conf on Dependable, Autonomic and Secure Computing, Intl Conf on Pervasive Intelligence and Computing, Intl Conf on Cloud and Big Data Computing, Intl Conf on Cyber Science and Technology Congress.

[66] VirusTotal. https://www.virustotal.com.

