# Supplementary material for: MINDPRES: A Hybrid Prototype System for Comprehensive Data Protection in the User Layer of the Mobile Cloud
Source: Sensors (Basel). 2025 Jan 23;25(3):670. doi: 10.3390/s25030670 (PMC11821073; doi:10.3390/s25030670)
Supplement: Supplementary file 1 [file sensors-25-00670-s001.zip › sensors-3336186-supplementary.pdf]

SUPPLEMENTARY MATERIAL: CLASs DIAGRAM, SEQUENCE DIAGRAM AND ACTIVITY DIAGRAM DESCRIPTIONS

MINDPRES: A Hybrid Prototype System for Comprehensive Data Protection in the User Layer of the Mobile Cloud

Noah Oghenefego Ogwara[1], Krassie Petrova[2]*, Mee Loong (Bobby) Yang[3] and Stephen G. MacDonell [4]

Copyright: © 2024 by the authors. Submitted for open access publication under the terms and conditions of the Creative Commons Attribution (CC BY) license (https://creativecommons.org/licenses/by/4.0/).

1 Auckland University of Technology; fego.ogwara@macvad.com
2 Auckland University of Technology; krassie.petrova@aut.ac.nz
3 Auckland University of Technology; bobby.yang@aut.ac.nz
4 Victoria University of Technology; stephen.macdonell@vuw.ac.nz
* Correspondence: krassie.petrova@aut.ac.nz

Table S1: Class diagram description

Class	Description
Main Activity	In the main activity class diagram, the setAppStateListener is a setter method that sets the values of the m_Listener attributes of the main activity class. Similarly, getAppUri maps the value of the URL retrieved by each app to the m_uri attributes of the main_activity class. The check_permission method evaluates the device's VPN permissions to allow the prototype system to either capture app activities or not. In addition, the captureServiceOk and captureServiceResults are responsible for capturing all API calls from all apps that reside on the device.
Device Manager	The device manager class depends on the main_activity class for some of its basic functionality. The OnCreate method of the device_manager class initiates the class activity and loads all the necessary data passed on by the main_activity class. The device_manager class sets up the VPN connection for the device and gets the device app list returned by the getAppList method. The device_manager class is also responsible for evaluating the user-installed apps on the device. It returns the total number of apps and device information via the getAppsCount and getDeviceID methods.
App Evaluator	The app_evaluator class also depends on the main_activity class to process the state of each resident app. The app_evaluator class inherits the methods in the AllAppData class and the permissionList class. The AllAppData class depends directly on the AppRiskModel class and AppRiskData class. The app_evaluator has five attributes that depend on the methods define d within its entity. The app_evaluator class executes the getRiskCategory of all apps and sets the app risk category for all user-installed apps in the device.. The outcomes depends on the results returned by the getMLPrediction method and getRiskScore

	method (these depend directly on other subclasses associated with the app_evaluator class)
Detection Engine	The detection_engine class depends on the main_activity class to get result of activities and URL request that were captured. The detection_engine class also inherits the AppOnlineActivities class that depend on the AllAppData class and AppActivites class. This enables the detection engine to execute the getAppOnlineActivites method that sets the values of Apps, TrafficData and TotalAppActivites attributes of the AppOnlineActivities class. These enable the detection engine to monitor the activities of all apps in the device.<br>The GetAllMaliciousActivites of the detection engines depends on the AppMaliciousActivities class that inherits methods in the AppActivites class;, which t depends directly on the URL Extractor class to set the malicious Activity attribute of the detection engine. Similarly, the GetAllBlacklistedActivites method of the detection engine also inherits methods of AppActivites class and the AppBlacklistedActivites class to set the attribute values of blacklistedActivites of the detection engine class. The detection engine also executes the enableBlacklistedActivites of the AppBlacklistedActivites class to allow a blacklisted activity to be active in the device.

Table S2: Sequence diagram description

Interaction	Description
Launch	The sequence diagram depicts the activity sequence of how the system interacts with each of the objects. First, when the system is launched, it gets the information about the device and prompts the user to grant the system access to the VPN service. The system will not monitor the activities of the apps if the user denies the system access to the VPN services.
VPN Access	Upon successful granting of access to the VPN services, MINDPRES sets up its own VPN connection that enables it to monitor the activities of all resident apps. First, the systems scans the apps and returns the total numbers of apps that reside on the device. Next, the app evaluator extracts the permission and intents demanded by each app and uses the information to evaluate the riskiness of each app and return a result.
Activity capture	The detection engine works in parallel with the device manage.. Once the VPN connection has been established the system starts capturing app activities. These activities are

	examined for possible intrusion. The activities deemed malicious by the detection engine are automatically blocked. The system also allows the user to re-activate blocked activities (in the case of a false alarm).

Table S3: Activity diagram description

Activity	Description
Launch and Initiate VPN	First, the user launches MINDPRES and initiates the VPN connection (the user needs to allow the system to access to the VPN service). The system automatically scans all apps on the device and sets up the VPN connection in the device manager activity class. The denial of a VPN connection by the user automatically stops the setting up of the VPN services. Hence, the system will not monitor the activities of the apps that reside on the device.
Evaluate	The user navigates between the three major activity tabs in the activity diagram. If the user selects the evaluate activity, the system automatically gets the list of all permissions and intents demanded by each app and executes two different activities simultaneously. The result from the two activities is used to determine the risk category of each app and the risk value associated with each of the apps that reside on the device.
Monitor	The selection of the detection engine gives the user the ability to navigate between three sub-activities associated with the detection engine. The default sub-activities are the app's online activities. These sub activities work in conjunction with the device manager and use the VPN services to retrieve all activities associated with each app that resides on the device. Id the app's malicious activities are selected, the system executes activities related to evaluating each app's traffic data and determining malicious connections. In addition, once a malicious activity is detected, the activity is blacklisted. The list of blacklisted traffic data is displayed on the user's screen.. This selection allows the user to either re-activate the blacklisted traffic data.
